# Global Methylome and gene expression analysis during early Peanut pod development

**DOI:** 10.1186/s12870-018-1546-4

**Published:** 2018-12-13

**Authors:** Pengfei Wang, Suhua Shi, Junjie Ma, Hui Song, Ye Zhang, Chao Gao, Chuanzhi Zhao, Shuzhen Zhao, Lei Hou, Javier Lopez-Baltazar, Shoujin Fan, Han Xia, Xingjun Wang

**Affiliations:** 1 Biotechnology Research Center, Shandong Academy of Agricultural Sciences; Shandong Provincial Key Laboratory of Crop Genetic Improvement, Ecology and Physiology, Jinan, 250100 People’s Republic of China; 2 Shandong Academy of Grape, Jinan, 250100 People’s Republic of China; 3 Life Science College of Shandong Normal University, Jinan, 250014 People’s Republic of China; 4 Life Science College of Shandong University, Jinan, 250100 People’s Republic of China; 5 Instituto Tecnologico del Valle de Oaxaca, 68000 Oaxaca, Mexico

**Keywords:** Peanut, Gynophore, Pod development, Methylome, Small RNA

## Abstract

**Background:**

Early peanut pod development is an important process of peanut reproductive development. Modes of DNA methylation during early peanut pod development are still unclear, possibly because its allotetraploid genome may cause difficulty for the methylome analysis.

**Results:**

To investigate the functions of the dynamic DNA methylation during the early development of the peanut pod, global methylome and gene expression analyses were carried out by Illumina high throughput sequencing. A novel mapping strategy of reads was developed and used for methylome and gene expression analysis. Differentially methylated genes, such as nodulin, cell number regulator-like protein, and senescence-associated genes, were identified during the early developmental stages of the peanut pod. The expression levels of gibberellin-related genes changed during this period of pod development. From the stage one (S1) gynophore to the stage two (S2) gynophore, the expression levels of two key methyltransferase genes, DRM2 and MET1, were up-regulated, which may lead to global DNA methylation changes between these two stages. The differentially methylated and expressed genes identified in the S1, S2, and stage 3 (S3) gynophore are involved in different biological processes such as stem cell fate determination, response to red, blue, and UV light, post-embryonic morphogenesis, and auxin biosynthesis. The expression levels of many genes were co-related by their DNA methylation levels. In addition, our results showed that the abundance of some 24-nucleotide siRNAs and miRNAs were positively associated with DNA methylation levels of their target loci in peanut pods.

**Conclusion:**

A novel mapping strategy of reads was described and verified in this study. Our results suggest that the methylated modes of the S1, S2, and S3 gynophore are different. The methylation changes that were identified during early peanut pod development provide useful information for understanding the roles of epigenetic regulation in peanut pod development.

**Electronic supplementary material:**

The online version of this article (10.1186/s12870-018-1546-4) contains supplementary material, which is available to authorized users.

## Background

Peanut is an important crop for oil and protein production in the tropic and subtropic regions worldwide. Peanut pod development is an important process of peanut reproductive development [1–3]. The peanut gynophore, the fertilized and elongated ovary, carries and eventually pushes the fertilized ovules into the soil where seed and pod development are completed [4, 5]. Within the aerial grown gynophore, pre-embryo development is arrested until the top region of the ovary is buried in the soil [4]. In early peanut embryo development, peanut endosperm can be observed [3, 6]. Similar to other dicotyledonous species, the endosperm is absorbed during the late developmental stages [7].

Previous studies indicated that light is the major inhibitor preventing embryo and pod development before the gynophore penetrates the soil [8, 9]. Light signals can be transduced through phytohormones to regulate plant growth and development, together with gibberellin (GA), abscisic acid (ABA), brassinosteroids, and ethylene [10–12]. Previous studies have indicated that phytohormones play important roles in the early development of peanut pods. For example, auxin affected the gravitropic growth of the gynophore [1]. However, the molecular events that regulate early peanut pod development remain unknown. DNA methylation is an important epigenetic marker and a conserved mechanism for gene regulation in both plants and animals [13]. DNA methylation modulates gene expression, controls invading viruses, silences transposable elements, and maintains genomic integrity in eukaryotes [14–16]. In plants, DNA methylation occurs at three sequence contexts, CG, CHG, and CHH [13, 17–19], and is mainly determined by both DNA methyltransferases and demethylases [16, 19]. In plants, there are two types of DNA methyltransferases for the maintenance of DNA methylation: methyltransferase (MET) and chromomethylase (CMT) [20, 21]. METs maintain CG methylation, while CMTs maintain CHH and CHG methylation [22, 23]. Domains Rearranged Methyltransferase (DRM) is the key enzyme for de novo DNA methylation and requires targeting information, which is often derived from small RNA pathways [22]. Pol IV derived long dsRNAs are processed by DICER-LIKE 3 (DCL3) into siRNAs and exported to the cytoplasm. Following the loading of one strand of siRNAs onto ARGONAUTE4 (AGO4), they are imported to nucleus, where siRNAs guide the targeting by sequence complementarity. Eventually, DNA methyltransferase is guided to target loci by AGO4 associated siRNAs [24].

DNA methylation and de-methylation, including repressor of silencing 1 (ROS1) and demeter (DME), are critical for Arabidopsis embryogenesis and seed viability [25]. Specifically, ROS1 induces DNA de-methylation in extensive tissues and DME preferentially induces DNA de-methylation in central cells [26]. The apical cell of the *met* mutant embryo divides incorrectly and the viability of the seed is reduced. Many embryo-specific genes are expressed abnormally in the *met* mutant embryo [27]. DNA methylation regulates the ripening process of tomato fruit through hypermethylation of the promoter of a colorless non-ripening gene [28].

The DNA methylation patterns of the whole peanut genome and the functions of DNA methylation on peanut pod development have not been reported. In this study, we analyzed DNA methylation of the peanut gynophore/pod at different developmental stages including (1) above ground green gynophores, (2) white gynophores that have been buried in soil for approximately 3 days, and (3) gynophores with a just enlarged pod that have been buried in soil for approximately 9 days. Dynamic DNA methylome and gene expression analyses of these three stages facilitated the investigation of the roles of DNA methylation on peanut pod development. Our results demonstrated that genes that may play key roles in embryogenesis are differentially methylated in these three developmental stages.

## Methods

### Plant materials

Peanut cultivar Luhua14 was used for this study. The criteria for gynophore/pod staging followed methods described in previous studies [29, 30]. Three millimeter tips of stage 1 (S1) and stage 2 (S2) gynophores and the enlarged ovary of the stage 3 (S3) gynophore were collected for DNA isolation.

### Genomic DNA extraction, library construction, and high throughput sequencing

Genomic DNA was extracted following a previously described method [31]. DNA was used for MeDIP libraries construction and each MeDIP library was subjected to high throughput sequencing by an Illumina solexa HiSeq2000 platform following previously described methods [31]. Two biological replicates were used for the DNA methylation analysis.

### Sequencing quality control and reads mapping

Quality control was performed using FastQC (http://www.bioinformatics.babraham.ac.uk/projects/fastqc/). Clean reads were mapped on the A and B sub-genome (pse-genomes) using Bowtie2 (version 2.0.5) software with default parameters [32]. Peanut pse-genome sequences were obtained from the peanut genome database (http://peanutbase.org/).

### The identification of orthologs between pse-a and B genomes

First, all protein coding genes of the pse-A and B genomes were identified by local all-vs.-all BLASTP (E-value < 10^− 20^). The best-hit gene-pairs between the pse-A and B genomes were selected for subsequent analysis. Secondly, interspecies synteny analysis of the pse-A or B genomes was based on comparisons of 100 kb chromosome blocks containing the genes of the best-hit gene-pairs [33, 34]. When three or more conserved homologous gene-pairs were individually detected in the two blocks from the pse-A and B genomes these two blocks were considered syntenic blocks [33–35], and the pairs of genes were considered orthologous between the pse-A and B genomes.

### Novel mapping strategy

Clean reads were mapped onto both the pse-A and B genomes. We first mapped one clean read to the pse-A genome, and then mapped the same read to the pse-B genome. Based on the mapping results, we could identify if this read could be mapped to pse-A, or pse-B, or both genomes. The mapping results were important for the next calculation of reads of genes per million mapped reads (RPM) of a particular gene. For this, we first confirmed whether the target gene is pse-A genome specific, pse-B genome specific, or present in both the pse-A and B genomes. For the pse-A or B genome specific genes, we counted the real number of reads that mapped to this gene for the calculation of RPM.

If the gene was present in both the pse-A and B genomes, it was considered orthologous for that target gene, and all reads that mapped to the orthologs of the pse-A and B genomes were counted for the calculation of RPM. However, two types of reads existed when they were mapped to the orthologs of the pse-A and B genomes. Specifically, type I reads could only be mapped to the orthologs of the pse-A or B genomes. If one read was type I, we used the real number of the read to calculate RPM. Type II reads could be mapped to both the orthologs of the pse-A and B genomes; therefore, one particular read could be counted twice. If the read was considered a type II read, it was counted only once when calculating the RPM of a particular gene, because if it was counted twice, the methylation level would have been overestimated.

### Identification of differentially methylated and expressed genes

The differentially expressed and methylated genes (t-test, *P* < 0.05) from peanut pod MEDIP-seq and RNA-seq datasets were identified based on RPM value using the software R (http://bioconductor.org/packages/release/bioc/html/edgeR.html). RPM was calculated as the clean read number mapped to the pse-A and B genomes of one gene per the total sequenced clean reads (millions). To identify the differentially expressed genes, we analyzed the RNA-seq data previously reported by Zhang (NCBI’s Short Read Archive under accession number SRP064700) [29]. The plant samples for this MEDIP-seq study were also same to previous study [29].

### Identification of peanut siRNA and their target loci in the pse-a and B genomes

Small RNAs were analyzed using our previously reported data [30]. The plant samples for this MEDIP-seq study were also same to previous study [30]. First, ribosomal RNAs, transfer RNAs, snoRNAs, and snRNAs were filtered to identify the siRNA candidates and miRNAs [30]. The TargetFinder software was used to identify the target loci in the pse-A and B genomes [36].

### Identification of DNA methylation related genes

The protein coding sequences of Arabidopsis RNA-directed DNA methylation (RdDM) pathway genes were used as queries to identify peanut RdDM pathway genes using BLASTP (E-value < 10^− 20^). Sequences with the smallest E-value and the highest identities were considered homologs of peanut genes.

## Results

### Strategy development for peanut DNA methylation and RNA-seq data analysis

Cultivated peanut is allotetraploid (AABB, 4n = 4x = 40), originating from a single hybridization event between the diploid A and B genomes from wild type species [35, 37–39]. Whole genome sequencing of the two ancestral species (*A. duranensis* and *A. ipaensis*) has been completed (http://peanutbase.org/) [40]. The cultivated peanut genome was sequenced but not assembled. The corrected median identities between cultivated peanut genome sequence reads and the pseudomolecules of *Arachis. duranensis* and *Arachis. ipaensis* were 98.36 and 99.96%, respectively [40]. In this study, we used *A. duranensis* and *A. ipaensis* as A and B sub-genome (pse-genomes) of cultivated peanut for sequence data analysis.

**Fig. 1:**
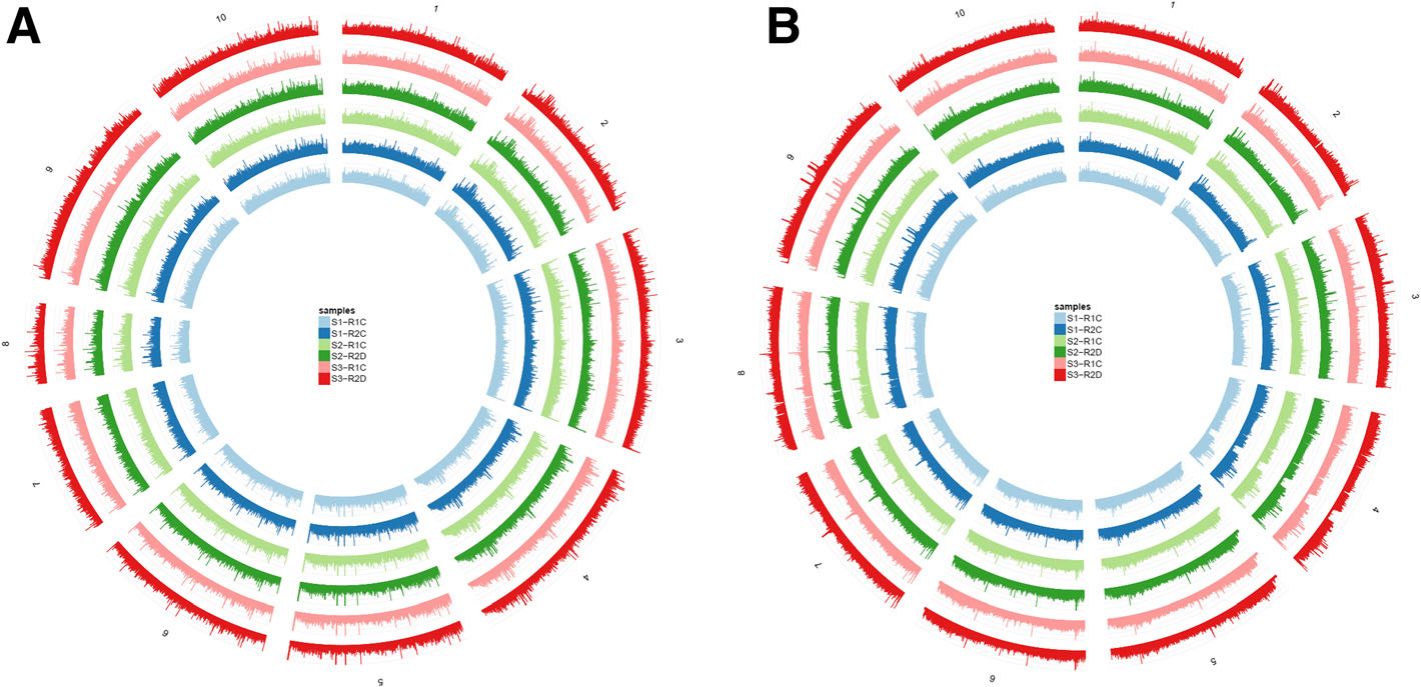
Distribution of DNA methylation in S1, S2 and S3 A and B genomes. **a** represents A genome, and **b** represents B genome. The height of circular coordinate axis represents DNA methylation depth

**Fig. 2:**
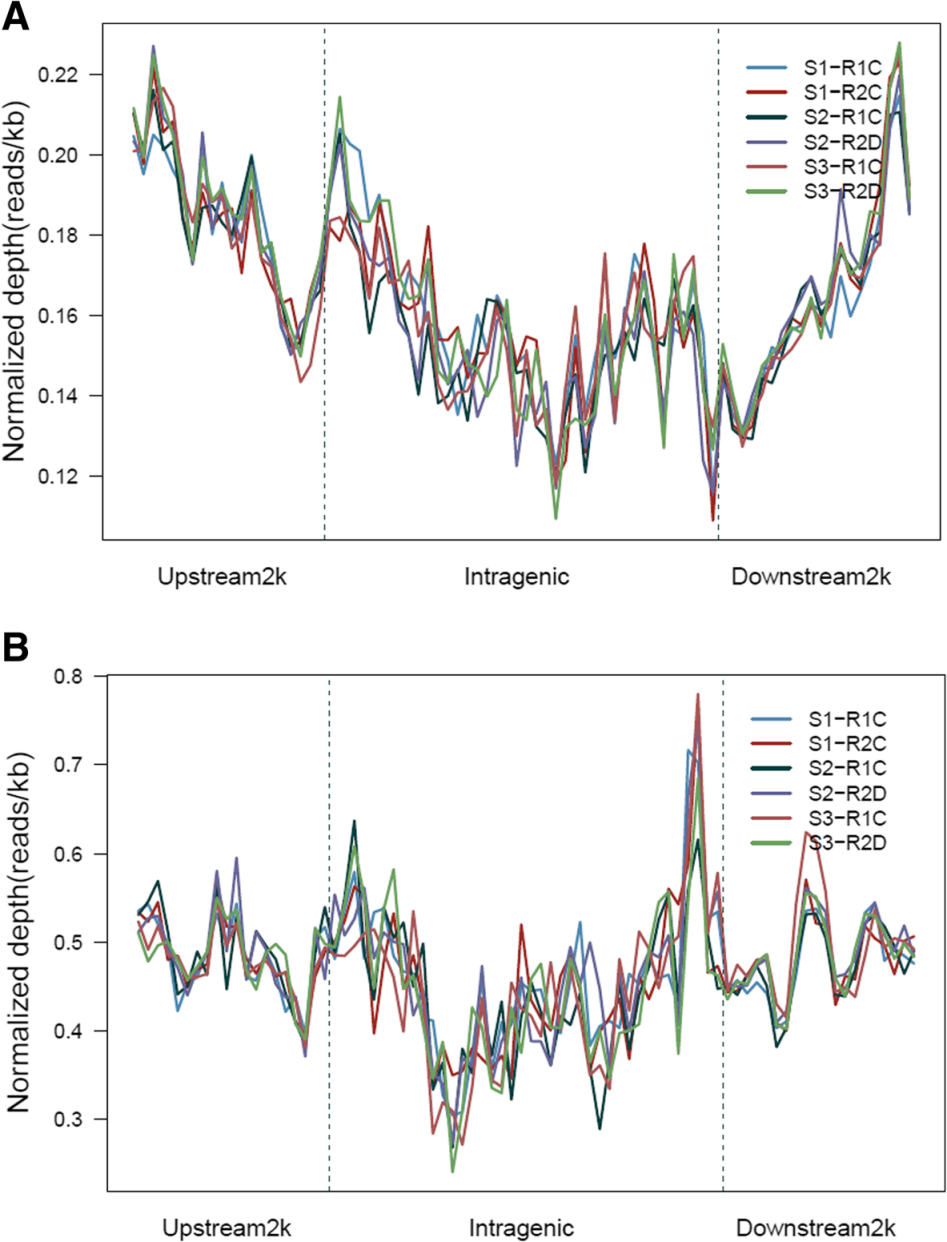
Distribution of DNA methylaion in S1, S2 and S3 genes from A and B genomes. **a** represents A genome, and **b** represents B genome. The height of coordinate axis represents t DNA methylation depth

**Fig. 3:**
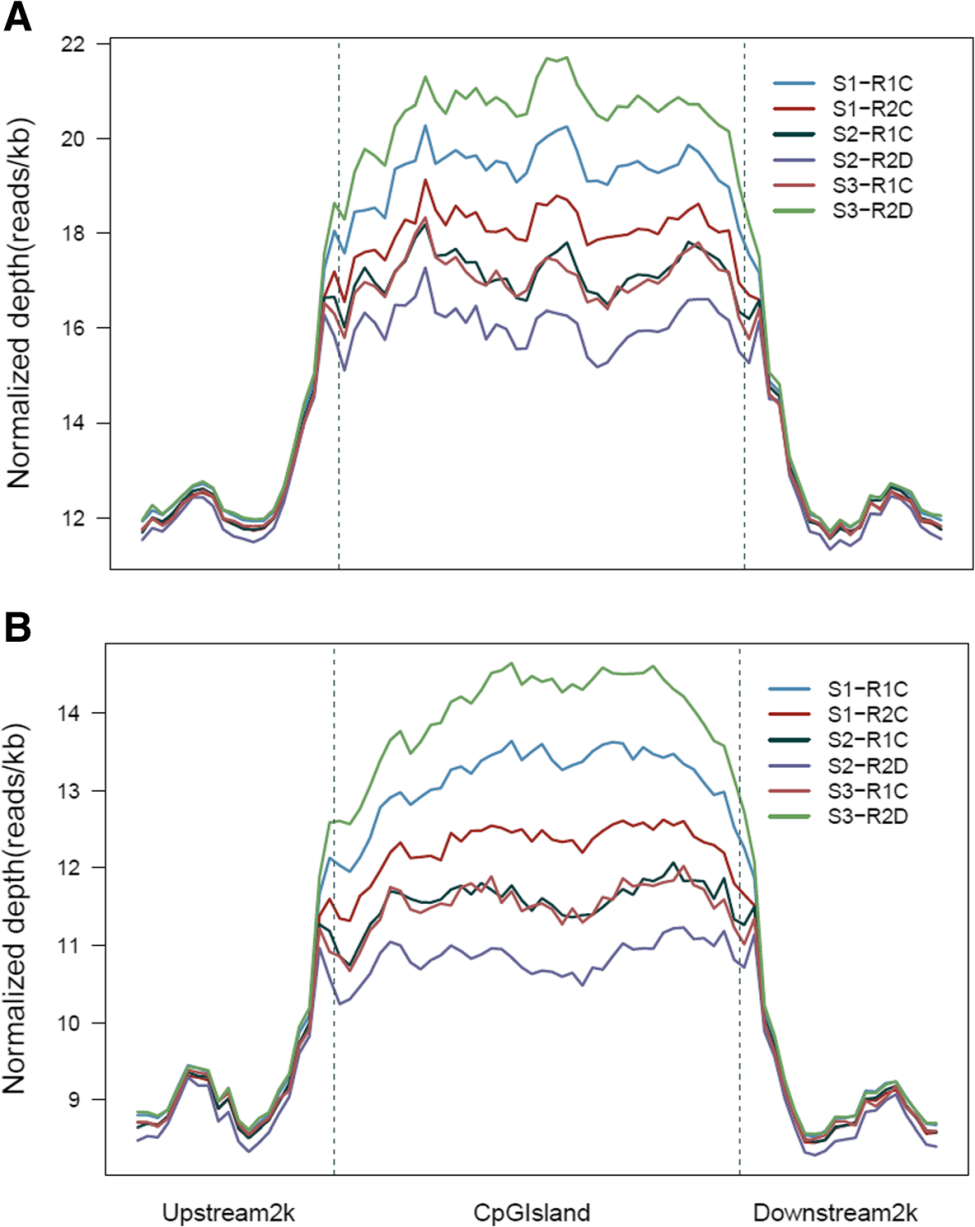
Distribution of DNA methylation in S1, S2 and S3 CpG island and CpG island shore from A and B genomes. **a** represents A genome, and **b** represents B genome. The height of coordinate axis represents the DNA methylation depth

Using the pse-A and pse-B genomes, the MEDIP-seq generated a total of approximately 42,857,143 clean reads from all samples (Short Read Archive in NCBI under accession number SUB4324289). MEDIP-seq data were mapped on pse-A and B genomes (Fig. 1). We found that the distribution of reads was different in gene coding regions compared with the promoter (upstream 2000 bp regions of genes) and transcript terminal regions (TTRs; downstream 2000 bp regions of genes) both in pse-A and pse-B genomes (Fig. 2). The CpG island was hyper-methylated compared with the CpG island shore (upstream and downstream 2000 bp regions of CpG island) both in pse-A and pse-B sub-genomes (Fig. 3).

When MEDIP-seq reads were mapped directly on the pse-A genome and pse-B genome, respectively, we encountered a problem. Reads were repeatedly mapped to the regions that were highly conserved between pse-A and B genomes, because of the existence of a large number of orthologous genes across these two sub-genomes. If the total MEDIP reads were mapped only to the A genome or only to B genome, the mapping rate was about approximately 79% because of the A or B genome specific genes. If we combined the reads mapped specifically to the pse-A or B genomes and to both genomes, the mapping rate increased to 95%. Therefore, our novel method improved the mapping strategy.

In this study, the gene methylation level was evaluated by RPM. Because we considered the orthologous genes as one gene, we had to count all reads that mapped to the orthologous genes located in both the pse-A or B genomes. The length of the orthologous genes could be different; therefore, we could use RPKM, unlike studies conducted in rice [41].

**Fig. 4:**
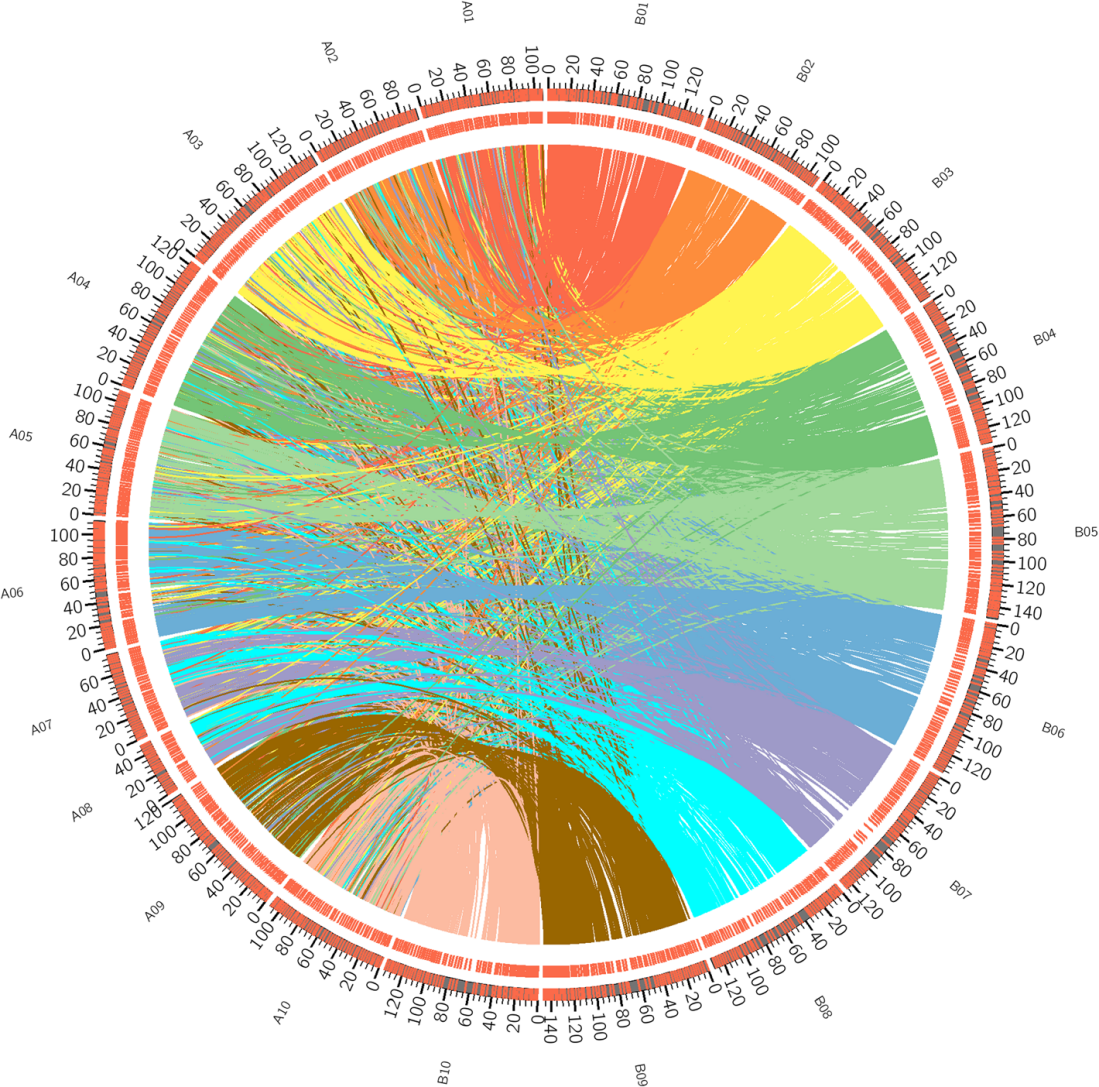
Distribution of orthologous-pairs in A and B genomes. Orthologous-pair members from A and B genomes were linked by color lines. A01-A10 represents chromosome 1–10 in A genome and B01-B10 represents chromosome1–10 in B genome

This study focused on the DNA methylation of protein-coding genes. The orthologous genes across the pse-A and B genome were considered alleles, because we presumed that orthologous genes within the same genus are functionally similar. Functional annotation of orthologous genes across pse-A and B genomes are listed in Additional file 1: Table S1. These DNA methylation or expression levels of orthologous genes were difficult to distinguish between the pse-A and B genomes. Likewise, DNA methylation or expression levels were also difficult to distinguish between paternal alleles and maternal alleles. However, the sum of DNA methylation of orthologous genes from the pse-A and B genome were determined. The non-orthologous genes from the A and B genomes were analyzed using the common method. We identified 40,639 and 46,985 protein-coding genes in the pse-A and B genomes, respectively, and 19,030 orthologous genes across the pse-A genome and B genomes (Additional file 1: Table S1; Fig. 4).

### Differential DNA methylation in protein-coding genes

Differentially methylated genes (DMG) were identified across the S1, S2, and S3 stages of the gynophores. There were 1055 DMGs between S1 and S2 including a large number of photosynthesis-related genes such as photosystem I assembly protein, photosystem II CP43 chlorophyll apoprotein, photosystem II D1, light-harvesting chlorophyll B-binding protein, and chlorophyll synthase genes. Cell division protein, cell wall protein expansin 2, and cell number regulator-like protein genes were differentially methylated in all three stages. WUSCHEL-related homeobox (WOX), nodulin, and growth regulating factor (GRF) zinc finger protein genes were identified as DMGs. The methylation levels of two senescence-associated genes were decreased in S2 and two AUX/IAA protein genes were increased in S2 compared with S1. The methylation levels of three ethylene-responsive transcription factor genes were down-regulated in S2 (Additional file 2: Table S2).

In total, 1190 DMGs were identified between S1 and S3, including cell division FtsZ-like protein, auxin response factor, auxin-responsive family protein, and GRF zinc finger protein genes. The methylation levels of four senescence-associated genes were all up-regulated in S3 (Additional file 2: Table S2). DNA methylation of the DME gene was down-regulated in S3 compared with S1.

The number of DMGs between S2 and S3 was 2207. Embryo defective genes were found to be differentially methylated. Genes involved in cell division or expansion, such as cell division cycle protein, cell wall protein EXP2, and cell number regulator-like protein genes, were differentially methylated. Twelve GRF zinc finger protein genes and five nodulin genes were identified as DMGs between S2 and S3. The methylation level of the PIF1 gene was down-regulated while the methylation level of the PIF3 gene was up-regulated in S3. A number of genes involved in hormone signal transduction, such as ethylene responsive transcription factor and auxin response factor genes, were identified as DMGs. DNA methylation levels of five senescence-associated genes increased in S3 (Additional file 2: Table S2).

**Fig. 5:**
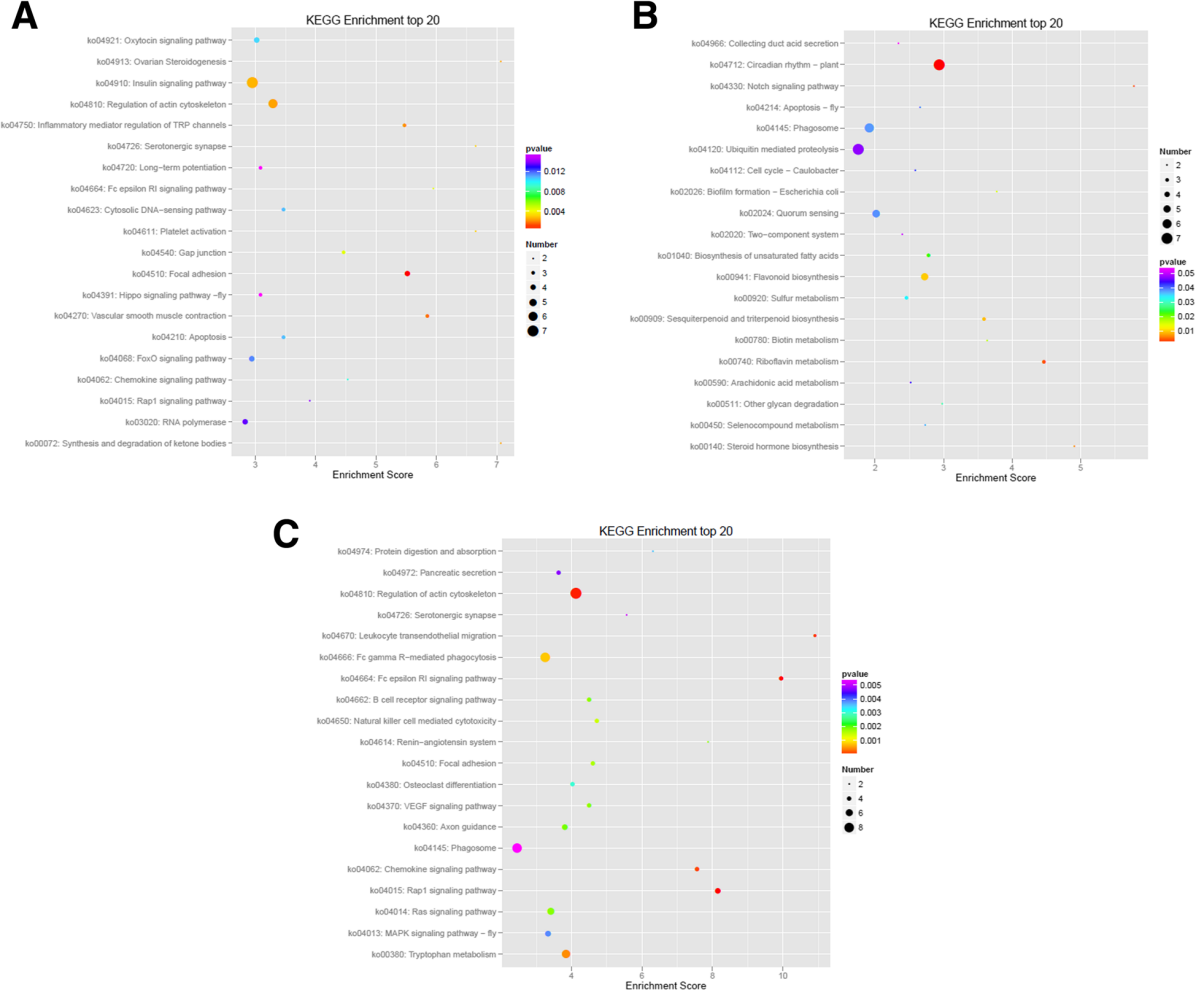
Top 20 enriched KEGG pathway of DMGs. **a** represents the top 20 enriched KEGG pathway of DMGs between S1 and S2 , **b** represents the top 20 enriched KEGG pathway of DMGs between S1 and S3, and **c** represents the top 20 enriched KEGG pathway of DMGs between S2 and S3. Color of the dots represents *P*-value, size of the dots represents the number of genes

KEGG pathway and enrichment analysis showed that DMGs were enriched (*P* < 0.05) in mRNA surveillance, oxidative phosphorylation, and regulation of actin cytoskeleton pathways between S1 and S2 (Fig. 5a; Additional file 3: Table S3). Some DMGs between S1 and S2 were involved in hormone signaling transduction. DMGs between S1 and S3 were enriched (*P *< 0.05) in ubiquitin mediated proteolysis, flavonoid biosynthesis, riboflavin metabolism, and sesquiterpenoid and triterpenoid biosynthesis (Fig. 5b; Additional file 3: Table S3). DMGs between S2 and S3 were enriched (*P* < 0.05) in regulation of actin cytoskeleton, RNA transport, cAMP signaling pathway, porphyrin and chlorophyll metabolism, tryptophan metabolism, and pentose phosphate pathway (Fig. 5c; Additional file 3: Table S3). Some DMGs between S2 and S3 were involved in hormone signaling transduction and cell cycle.

### Mapping and analysis of RNA-seq data

Using the improved analysis strategy, we analyzed RNA-seq data previously published by Zhang (NCBI accession number SRP064700) [29]. Results showed that the reads mapping rate of these sequences increased from 70 to 95% using our new strategy.

Many differentially expressed genes (DEGs) were found across the three developmental stages of gynophores. In total, 2337 DEGs were identified between gynophores in S1 and S2. Many photosynthesis-related genes were down-regulated in S2 including photosystem I reaction center subunit X, light-harvesting chlorophyll B-binding protein, and photosystem II reaction center protein genes. Two senescence-associated proteins, gibberellin 20 oxidase 1, and gibberellin 20 oxidase 2 genes were down-regulated in S2. Auxin response factor 4 and auxin transporter-like protein 5-like isoform X1 genes were also down-regulated in S2. Many ethylene-responsive transcription factor genes and three cell wall protein genes were up-regulated in S2 (Additional file 4: Table S4).

Between S1 and S3, 3169 DEGs were identified. Two differentially expressed ABA receptor genes were down-regulated in S3, and many hormone (ethylene, gibberellin, and auxin) signal transduction related genes were differently expressed between S1 and S3. Many nodulin, nodulin-like, and WOX genes were differentially expressed between S1 and S3 (Additional file 4: Table S4). Seven nodulin genes were up-regulated in S3, and many WOX and hormone signal transduction related genes were differentially expressed between S1 and S3. Two differentially expressed ABA receptors were down-regulated in S3 (Additional file 4: Table S4). Between S2 and S3, 1849 DEGs were discovered.

### Correlation between expression and DNA methylation

**Fig. 6:**
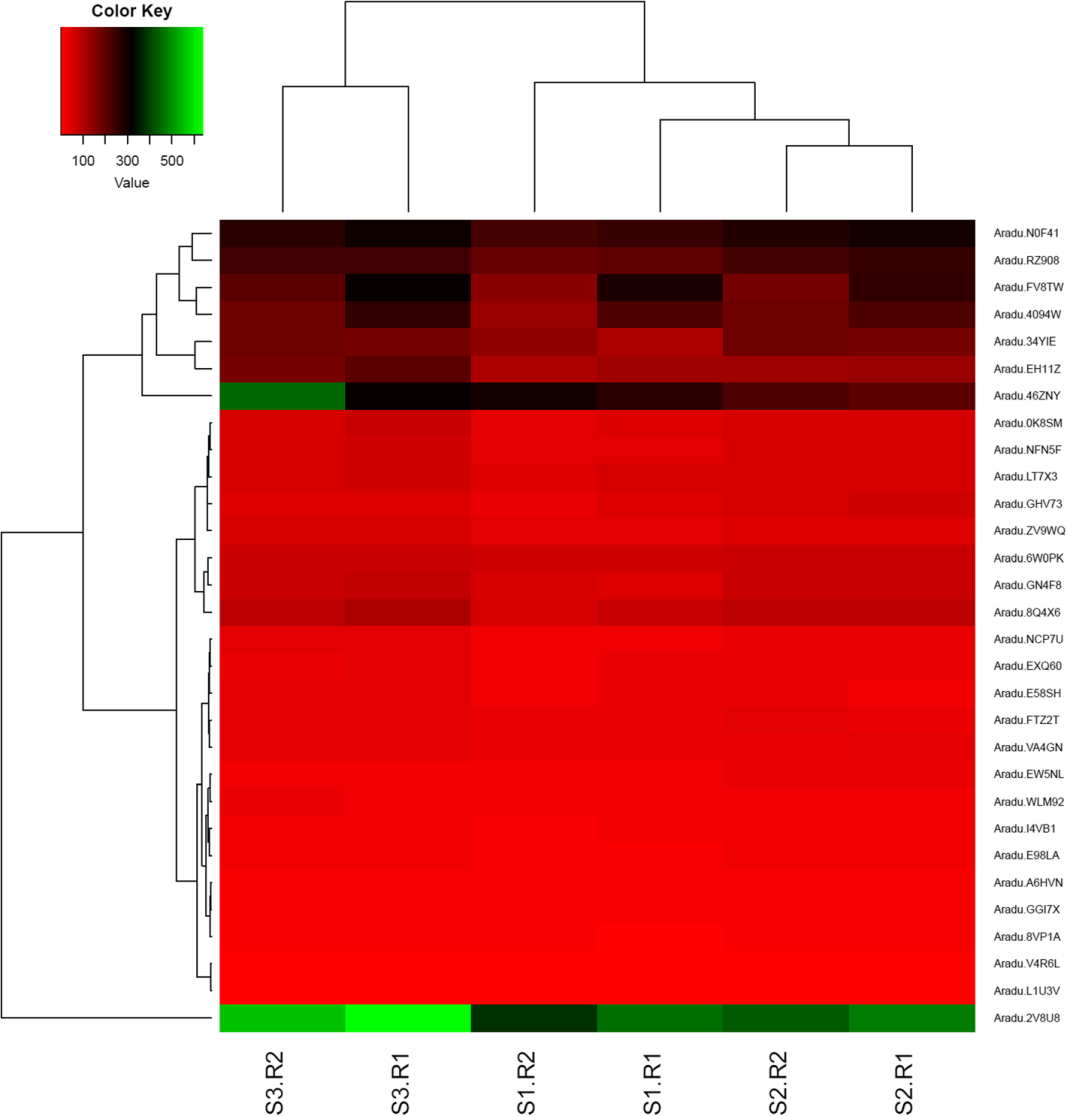
Expression heatmap of DNA methylation-related genes in S1, S2 and S3

RdDM is the major small RNA-mediated epigenetic pathway in plants. The dynamic expression of genes in the RdDM pathway was analyzed (Table 1). We found that the *AGO6* (Aradu.E98LA), *DRM2*, *MET1*, and *DEFECTIVE IN MERISTEM SILENCING 3* (*DMS3*) were all up-regulated in S2 compared with the expression in S1 (Fig. 6).

**Table 1:** DNA methylation-related genes in peanut

	Orthologous from	Orthologous from	Orthologs in
Gene name	A genome	B genome	Arabidopsis
NUCLEAR RNA POLYMERASE D1(NRPD1)	Aradu.EXQ60	Araip.NHV7F	AT1G63020
NRPE1	Aradu.U7301	Araip.A5H9W	AT2G40030
NRPD2/NRPE2	Aradu.8Q4X6	Araip.AVR8T	AT3G23780
NRPD4/NRPE4	Aradu.WLM92	Araip.F0FD4	AT4G15950
NRPE5	Aradu.LT7X3	Araip.2H1ED	AT3G57080
NRPE9B	Aradu.8VP1A	Araip.4786 J	AT4G16265
NRPB1	Aradu.FV8TW	Araip.AAM5W	AT4G35800
RNA-DEPENDENT RNA POLYMERASE 2 (RDR2)	Aradu.0K8SM	Araip.63JS7	AT4G11130
DICER-LIKE 3 (DCL3)	Aradu.EH11Z	Araip.0XV9H	AT3G43920
HUA ENHANCER 1 (HEN1)	Aradu.I4VB1	Araip.0P57Y	AT4G20910
ARGONAUTE 4 (AGO4)	Aradu.2V8U8	Araip.DM5GK	AT2G27040
AGO6	Aradu.E98LA	Araip.70L1G	AT2G32940
AGO9	Aradu.2V8U8	Araip.DM5GK	AT5G21150
CLASSY 1 (CLSY1)	Aradu.TP9JP	Araip.VW316	AT3G42670
DEFECTIVE IN RNA-DIRECTED DNA METHYLATION 1 (DRD1)	Aradu.E58SH	Araip.VVZ0N	AT2G16390
DEFECTIVE IN MERISTEM SILENCING 3 (DMS3)	Aradu.NCP7U	Araip.X02G0	AT3G49250
RNA-DIRECTED DNA METHYLATION 1 (RDM1)	Aradu.0VT9B	Araip.6D36H	AT3G22680
KOW DOMAIN-CONTAINING TRANSCRIPTION FACTOR 1 (KTF1)	Aradu.4094 W	Araip.J0JMG	AT5G04290
INVOLVED IN DE NOVO 2 (IDN2)	Aradu.RZ908	Araip.15C6I	AT3G48670
IDN2 PARALOGUE 1 (IDP1)	Aradu.N0F41	Araip.47FQ5	AT1G15910
IDP2	Aradu.N0F41	Araip.47FQ5	AT4G00380
DMS4	Aradu.EW5NL	Araip.C9F8X	AT2G30280
DOMAINS REARRANGED METHYLTRANSFERASE 2 (DRM2)	Aradu.NFN5F	Araip.B5M84	AT5G14620
SUVH2	Aradu.A6HVN	Araip.M3BFQ	AT2G33290
SUVH9	Aradu.A6HVN	Araip.M3BFQ	AT4G13460
SUVR2	Aradu.VA4GN	Araip.1D0HT	AT5G43990
MICRORCHIDIA 1 (MORC1)	Aradu.FTZ2T	Araip.B1U9G	AT4G36290
MORC6	Aradu.FTZ2T	Araip.B1U9G	AT1G19100
SAWADEE HOMEODOMAIN HOMOLOGUE 1 (SHH1)	Aradu.GGI7X	Araip.U8UTV	AT1G15215
HISTONE DEACETYLASE 6 (HDA6)	Aradu.L1U3V	Araip.WB197	AT5G63110
JUMONJI 14 (JMJ14)	Aradu.V4R6L	Araip.Z2DN9	AT4G20400
LYSINE-SPECIFIC HISTONE DEMETHYLASE 1 (LDL1)	Aradu.ZV9WQ	Araip.F5V5K	AT1G62830
LDL2	Aradu.9QT6G	Araip.M4152	AT3G13682
UBIQUITIN-SPECIFIC PROTEASE 26 (UBP26)	Aradu.A0F5T	Araip.R2ZRV	AT3G49600
NEEDED FOR RDR2.INDEPENDENT DNA METHYLATION (NERD)	Aradu.EB2 7 U	Araip.FJ8IU	AT2G16485
CHROMOMETHYLASE 2 (CMT2)	Aradu.6W0PK	Araip.71XM6	AT4G19020
CMT3	Aradu.34YIE	Araip.527SE	AT1G69770
METHYLTRANSFERASE 1 (MET1)	Aradu.GN4F8	Araip.RYZ61	AT5G49160
SUVH4	Aradu.I17CD	Araip.A9GQY	AT5G13960
DECREASED DNA METHYLATION 1 (DDM1)	Aradu.LCP0L	Araip.5QZ4M	AT5G66750
CHROMOMETHYLASE 2 (CMT2)	Aradu.6W0PK	Araip.71XM6	AT4G19020
METHYLTRANSFERASE 1 (MET1)	Aradu.GN4F8	Araip.RYZ61	AT5G49160
DME	Aradu.GHV73	Araip.81XFD	AT5G04560

Compared with the expression in S1, *DEFECTIVE IN RNA-DIRECTED DNA METHYLATION 1* (*DRD1*), *MICRORCHIDIA 1* (*MORC1*), and *INVOLVED IN* DE NOVO *2* (*IDN2*) were all up-regulated in S3 (Fig. 6).

*LYSINE-SPECIFIC HISTONE DEMETHYLASE 1* (*LDL1*) was expressed at the highest level in S3 compared with that in S1 and S2. The expression of *LDL1* between S1 and S3 as well as S2 and S3 were different, while DNA methylation levels of this gene were different only between S1 and S2. DNA methylation levels of most genes in the RdDM pathway were stable across three stages except *NRPE5*, *HISTONE DEACETYLASE 6* (*HDA6*), *LDL1*, and *DMS3*. The expression levels of other demethylase genes were stable in the development of the gynophores from S1to S3 (Fig. 6).

### Differentially methylated and expressed genes (DMEGs)

Between S1 and S2, 69 DMEGs were detected, including photosystem I assembly protein, light-harvesting chlorophyll B-binding protein, and photosystem II protein D1 genes, which are all related to photosynthesis. One nodulin gene, one Dof zinc finger protein gene, and one ICE1-like transcription factor were DMEGs. Some DMEGs between S1 and S2 were involved in disease resistance such as disease-resistance response protein (Additional file 5: Table S5). DMEGs between S1 and S2 were enriched in stem cell fate determination, root development, lignin biosynthetic process, response to temperature stimulus, red light, blue light, and UV light (Additional file 6: Table S6).

Between S1 and S3, 117 DMEGs were detected, including photosystem I reaction center subunit IV, photosystem II CP43, and chlorophyll apoprotein genes. Some DMEGs between S1 and S3 were involved in hormone signal transduction, such as auxin-responsive family protein genes. AP2-like ethylene-responsive transcription factor and bHLH transcription factor genes were DMEGs between S1 and S3 (Additional file 5: Table S5). DMEGs between S1 and S3 were enriched in post-embryonic morphogenesis, organ boundary specification between lateral organs and meristem, auxin biosynthesis, and cell wall organization (Additional file 6: Table S6).

Between S2 and S3, 61 DMEGs were detected, including some photosynthesis related genes, germin-like protein, LRR, and NB-ARC domain disease resistance genes (Additional file 5: Table S5). DMEGs between S2 and S3 were enriched in embryo development, cold acclimation, regulation of cell division, seed coat development, embryo sac development, and post-embryonic morphogenesis (Additional file 6: Table S6).

### The relationship between gene DNA methylation and expression levels

Most protein-coding genes were stable both in their expression and methylation across the three developmental stages. In the 69 identified DMEGs between S1 and S2, 24 were differentially methylated in the promoter region, 22 in the gene body, and 22 in the TTR. Among them, the expression of 29 genes (42.03%) was negatively related to their DNA methylation level. Ten of these 29 DMEGs were differentially methylated in the promoter region, eight in gene body, and 11 in the TTR (Additional file 5: Table S5). In 117 DMEGs between S1 and S3, 39 were differentially methylated in the promoter, 47 in the gene body, and 31 in the TTR. The expression of 47 genes (40.17%) was negatively related to their DNA methylation level. Among these 47 DMEGs, 11 genes were differentially methylated in the promoter, 26 in the gene body, and 10 in the TTR (Additional file 5: Table S5). In the 61 DMEGs between S2 and S3, 24 were differentially methylated in the promoter, 17 in the gene body, and 20 in TTR. The expression of 21 genes (34.43%) was negatively related to the DNA methylation level. Among these 21 DMEGs, nine genes were differentially methylated in the promoter, six in the gene body, and six in the TTR (Additional file 5: Table S5).

### The relationship between small RNA level and DNA methylation

We analyzed the relationship between the abundance of 24-nucleotide (nt) siRNAs and the DNA methylation level of their target loci in peanut gynophores using previously reported data from small RNA in three stages of gynophore development [30] (Additional file 7: Table S7). The abundance of some 24-nt siRNAs was indeed positively associated with the DNA methylation level of some target loci such as siRNA t0207414 and t0470617. Twenty 24-nt siRNAs were not expressed in S3 but expressed in S1, and 30 of their target loci DNA methylation levels were decreased in S3. Thirteen 24-nt siRNAs were not expressed in S2 but were expressed in S1, and 45 of their target loci had decreased DNA methylation levels in S2. The relationship between the abundance of miRNA and DNA methylation level of their target loci was investigated using our previously reported miRNA data [30]. A positive relationship was observed between the abundance of some miRNAs and target loci methylation such as peanut miR5713, miR2108b, and miR8762d.

## Discussion

In wheat, the methylome sequencing reads were mapped onto the diploid ancestor sub-genome A, B, and D individually [42–44], using SNPs to distinguish three sub-genomes [42]. When this strategy was used in peanut, we could only detect the methylated regions of DNA where there are SNPs and the general DNA methylation level of the sub-genome. This method missed DNA methylation information from genes with no SNPs between the sub-genomes. Additionally, SNPs only covered part of the genome, and many clean reads not located near the SNP regions were filtered out. Thus, to improve the coverage, we combined the reads mapped specifically to the pse-A or B genomes and to both genomes, increasing the mapping rate of each sample (both MEDIP-seq and RNA-seq data) to 95%. Here, a novel strategy was used. We considered the orthologs as one gene and counted all reads that mapped to the orthologs of the pse-A and B genomes for the calculation of RPM. Therefore, the DNA methylation levels of nearly all peanut protein-coding genes could be examined. However, using this strategy, we could only use the RPM value to analyze the dynamic DNA methylation of the same gene in different samples. DNA methylation levels could not be compared between different genes.

In the development of gynophores from S1 to S3, the DNA methylation levels of many genes varied. From S1 to S2, the expression levels of two key methyltransferase genes, DRM2 and MET1, were up-regulated. The increased expression of these genes may lead to DNA methylation changes from S1 to S2 gynophores, which experienced environmental change from light to dark conditions. A previous study showed that light could affect DNA methylation of some genes, such as maize PEPC genes [45]. S1 and S2 gynophores grow in the light and dark, respectively, and the methylation levels of many genes changed during these two stages. However, the methylation level of peanut PEPC genes remained unchanged between S1 and S2 gynophores. Many genes involved in cell division, expansion, ethylene, and auxin signal transduction were considered DMGs in S1 and S2 gynophores. During S2 and S3, many genes involved in cell division, expansion, senescence, nodulin, ethylene, and auxin signal transduction were also DMGs that may be associated with embryo and pod development. DNA methylation levels of many GRF transcription factor genes involved in fruit development [46] were higher in S3 compared to S2 gynophores. Interestingly, DNA methylation levels of PIF1 and PIF3 genes, key components of light signal transduction involved in hypocotyl elongation and other developmental processes [47], varied in S2 and S3. DNA methylation affected genes involved in multiple biological processes, suggesting that DNA methylation may play a key role in early peanut pod development.

Previous studies have shown that DNA methylation could repress gene transcription [45, 48]. DNA methylation could also be positively related to gene expression [49–51]. In our study, we found that the expression levels of many genes were positively or negatively related to DNA methylation levels, while the expression levels of many other genes were not correlated with their methylation levels. The expression of some auxin and ethylene-related genes were correlated with their DNA methylation levels, and both of these hormones play important roles in peanut pod development [29]. In addition, gibberellins play important roles in early peanut pod development [29]. Similarly, the expression levels of many gibberellin-related genes varied among S1, S2, and S3; however, the DNA methylation levels of these gibberellin-related genes were stable. A previous study showed that ABA was very important for early peanut pod development [29]. Our results showed that ABA-regulated genes were enriched in DMEGs of S1 and S3. In other words, the expression of many ABA-regulated genes was correlated with their methylation levels.

Walls are thin 1 (WAT1), an Arabidopsis homolog of nodulin MtN21, is required for secondary wall formation [52]. Nodulin MtN21 is involved in nodule development [53]. A previous study indicated that a senescence-associated nodulin gene is a candidate for embryo abortion of aerial pods [54]. The expression levels of many nodulin genes varied in S1, S2, and S3, and the expression levels of some nodulin genes were correlated with their methylation levels. A previous study showed that high expression of senescence-associated genes might lead to embryo abortion. For example, the gynophores growing in continuous light express a high level of senescence-associated genes and cannot form pods. Down-regulation of these genes under dark conditions could be a critical factor for the initiation of embryo and pod development [54]. Our RNA-seq results showed that one senescence-associated gene was down-regulated in S2 compared to S1. However, the expression of this gene was not correlated with its methylation level.

Small RNA is an important trigger of the RdDM pathway [24]. Small RNA could affect embryo or seed development by DNA methylation. For example, small RNA could be transported from the endosperm to the embryo leading to embryo hypermethylation in rice [55]. A previous study showed that 24-nt siRNAs play a major role in the RdDM pathway [24]. A recently conducted study showed that DNA methylation of targets depended on miRNAs [56]. Our results are in agreement with this recent study, confirming that the abundance of 24-nt siRNAs and miRNAs were indeed positively associated with DNA methylation of their target loci in peanut pods.

## Conclusions

The improved mapping strategy could efficiently map reads to allotetraploid peanut DNA, increasing the mapping rate from 79 to 95%. MeDIP-seq data indicated that the methylation varied among the S1, S2, and S3 gynophores. The methylation levels of some genes differed in S1 to S3, which could be associated with the expression level of these genes. Genes involved in cell division, expansion, senescence, nodulin, ethylene, and auxin signal transduction were among the DMGs and may be associated with embryo development and pod formation. The expression levels of many genes involved in pod expansion varied in S1, S2, and S3. The senescence-related and nodulin genes may play important roles in peanut pod development. The change of the methylome during early peanut pod development is useful for understanding the molecular mechanisms that regulate peanut pod development.

## Additional files

Additional file 1: **Table S1.** Orthologous genes from peanut A and B sub-genome. (XLS 3611 kb)

Additional file 2: **Table S2.** Differential DNA methylation in protein-coding genes. (XLS 8009 kb)

Additional file 3: **Table S3.** Enriched KEGG pathway of DMGs. (XLS 35 kb)

Additional file 4: **Table S4.** Differentially expressed genes. (XLS 8234 kb)

Additional file 5: **Table S5.** Differentially methylated and expressed genes. The type “Exp” represent information of gene expression, and “Meth” represent information of gene DNA methylation. (XLS 115 kb)

Additional file 6: **Table S6.** Enriched GO term of DMEGs. (XLS 86 kb)

Additional file 7: **Table S7.** siRNA expressed in three stages. (XLS 1247 kb)

## Abbreviations

ABA: Abscisic acid
bHLH: Basic helix-loop-helix
CMT: Chromomethylase
DEG: Differentially expressed gene
DME: Demeter
DMEG: Differentially methylated and expressed gene
DMG: Differentially methylated genes
DMS3: DEFECTIVE IN MERISTEM SILENCING 3
DRD1: DEFECTIVE IN RNA-DIRECTED DNA METHYLATION 1
DRM: Domains Rearranged Methyltransferase
GA: Gibberellin
IDN2: INVOLVED IN DE NOVO 2
LDL1: LYSINE-SPECIFIC HISTONE DEMETHYLASE 1
MET: Methyltransferase
MORC1: MICRORCHIDIA 1
RdDM: RNA-directed DNA methylation
ROS1: Repressor of silencing 1
RPM: MEDIP-seq reads of gene per million mapped reads
